# Limitations of the human iPSC-derived neuron model for early-onset Alzheimer’s disease

**DOI:** 10.1186/s13041-023-01063-5

**Published:** 2023-11-03

**Authors:** Phoebe Valdes, Kenneth W. Henry, Michael Q. Fitzgerald, Koushik Muralidharan, Andrew B. Caldwell, Srinivasan Ramachandran, Lawrence S. B. Goldstein, William C. Mobley, Douglas R. Galasko, Shankar Subramaniam

**Affiliations:** 1 Department of Bioengineering, University of California, La Jolla, San Diego, CA USA; 2 Bioengineering Graduate Program, University of California, La Jolla, San Diego, CA USA; 3 Department of Cellular and Molecular Medicine, University of California, La Jolla, San Diego, CA USA; 4 Medical Scientist Training Program, University of California, La Jolla, San Diego, CA USA; 5 School of Medicine, University of California, La Jolla, San Diego, CA USA; 6 Sanford Stem Cell Clinical Center, University of California, La Jolla, San Diego, CA USA; 7 Department of Neurosciences, University of California, La Jolla, San Diego, CA USA; 8 Department of Nanoengineering, University of California, La Jolla, San Diego, CA USA; 9 Department of Computer Science and Engineering, University of California, La Jolla, San Diego, CA USA; 10 Present Address: Sanford Consortium for Regenerative Medicine, La Jolla, CA USA

**Keywords:** Early-onset Alzheimer’s disease, iPSC neurons, RNA-seq, Systems biology

## Abstract

Non-familial Alzheimer’s disease (AD) occurring before 65 years of age is commonly referred to as early-onset Alzheimer’s disease (EOAD) and constitutes ~ 5–6% of all AD cases (Mendez et al. in Continuum 25:34–51, 2019). While EOAD exhibits the same clinicopathological changes such as amyloid plaques, neurofibrillary tangles (NFTs), brain atrophy, and cognitive decline (Sirkis et al. in Mol Psychiatry 27:2674–88, 2022; Caldwell et al. in Mol Brain 15:83, 2022) as observed in the more prevalent late-onset AD (LOAD), EOAD patients tend to have more severe cognitive deficits, including visuospatial, language, and executive dysfunction (Sirkis et al. in Mol Psychiatry 27:2674–88, 2022). Patient-derived induced pluripotent stem cells (iPSCs) have been used to model and study penetrative, familial AD (FAD) mutations in *APP*, *PSEN1*, and *PSEN2* (Valdes et al. in Research Square 1–30, 2022; Caldwell et al. in Sci Adv 6:1–16, 2020) but have been seldom used for sporadic forms of AD that display more heterogeneous disease mechanisms. In this study, we sought to characterize iPSC-derived neurons from EOAD patients via RNA sequencing. A modest difference in expression profiles between EOAD patients and non-demented control (NDC) subjects resulted in a limited number of differentially expressed genes (DEGs). Based on this analysis, we provide evidence that iPSC-derived neuron model systems, likely due to the loss of EOAD-associated epigenetic signatures arising from iPSC reprogramming, may not be ideal models for studying sporadic AD.

**Supplementary Information:**

The online version contains supplementary material available at 10.1186/s13041-023-01063-5.

## Results and discussion

**Fig. 1:**
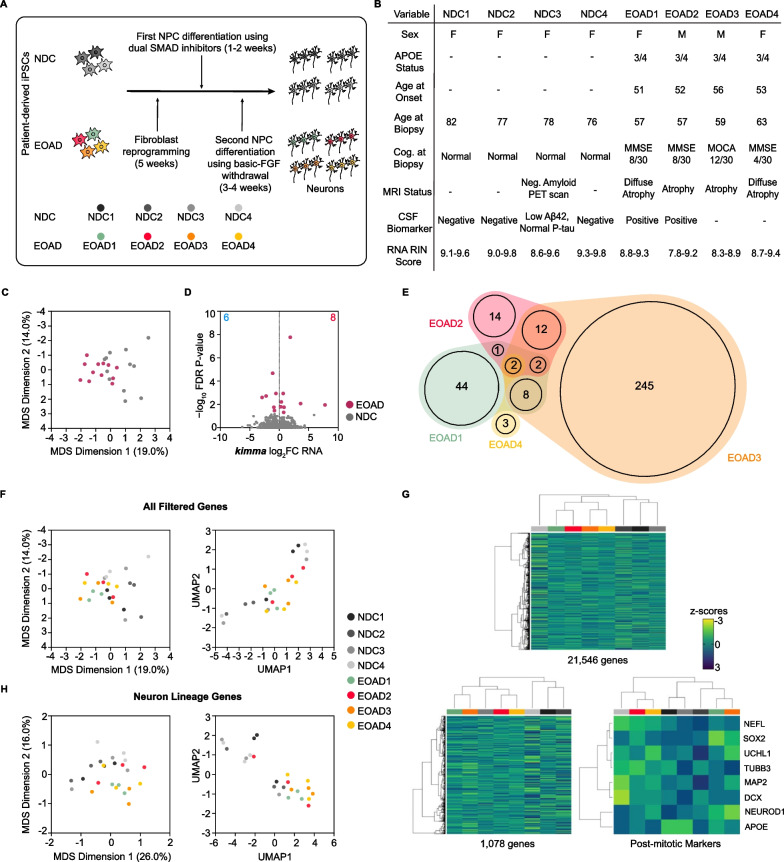
**A** Non-demented controls (NDCs) and EOAD iPSCs were differentiated using dual SMAD inhibitors (first step) and basic-FGF withdrawal (second step). **B** Metadata for NDC and EOAD subjects used in this study. **C** Multi-dimensional scaling (MDS) analysis after batch correction by experimental condition, sex and sequencing batch of filtered normalized RNA-seq data. **D** RNA-seq volcano plot of differentially expressed genes (DEGs) for EOAD patients relative to all NDCs as determined by *kimma* with an FDR p-value < 0.05. **E**  Quasi-proportional Venn diagram overlap of DEGs across the four EOAD patients relative to all NDC subject neurons. **F** MDS (left) and UMAP (right) analysis based on NDC and EOAD neurons generated in this study using filtered, normalized RNA-seq data. **G** RNA expression profile heatmap corresponding to all filtered genes. **H** MDS (left) and UMAP (right) analysis based on NDC and EOAD neurons generated in this study using filtered, normalized RNA-seq data for neuron lineage genes. **I** RNA expression profile heatmap corresponding to either neuron lineage genes (left) or post-mitotic genes (right) clustered by subject experimental condition (NDC or EOAD)

In this work, we generated induced pluripotent stem cell (iPSC)-derived neurons from 4 non-familial, early-onset Alzheimer’s disease (EOAD) patients (age at onset (AAO) 51–56 years) and 4 non-demented control (NDC) subjects whose age at biopsy was 76–82 years. Samples were provided by the UC San Diego Alzheimer’s Disease Research Center (ADRC). Individual clones (*n* = 3) from all 8 subjects were transformed into iPSCs and differentiated into neurons as previously described [6]. RNA was extracted from frozen neuron pellets (Fig. 1A). All 4 EOAD patients displayed diffuse cerebral atrophy by Magnetic Resonance Imaging (MRI) and decreased cognition as evidenced by either Mini-Mental State Examination (MMSE) scores less than 15 [7] or Montreal Cognitive Assessment (MoCA) score less than 25 [8], indicating a moderate to an advanced stage of EOAD progression (Fig. 1B). To the best of our knowledge, this is the first report using iPSC-derived neurons to model non-familial EOAD. RNA-seq was used to characterize gene expression dysregulation in EOAD and assess possible underlying mechanisms.

RNA isolated from EOAD and NDC neurons were of high quality, with RNA Integrity Numbers (RIN) ranging from 7.8 to 9.8. We assessed how the neurons from EOAD patients clustered with respect to NDC subjects in multi-dimensional scaling (MDS) space following correction for sex, sequencing batch, and experimental condition. This revealed no clear clustering of NDC or EOAD samples but rather a slight overlap between them (Fig. 1C). This may not be surprising, as EOAD displays heterogeneity in clinical presentation [2]. Differential expression analysis between two conditions revealed a small number of differentially expressed genes (DEGs) (*n* = 14) using the same covariates in the linear design model (Fig. 1D). To determine whether heterogeneity between the EOAD patients was causing the low number of DEGs, we compared each EOAD relative to all control subjects. While EOAD patient #3 displayed a higher amount of DEGs relative to all NDC subjects (n = 323), all other individual EOAD patients demonstrated a similar magnitude of DEGs (Additional file 1: Fig. S3A) with only 2 common DEGs among them (Fig. 1E). Next, we sought to assess the variance within individual clones of the same subject, regardless of EOAD or NDC, in MDS space for all filtered genes; we observed sparse grouping within each subject (less in EOAD but more in NDC) and slight overlap between the two groups (Fig. 1F, left). To assess whether a dimension reduction approach would reveal a better separation of EOAD and NDC neurons, we performed UMAP on the normalized, filtered counts. Here, we observed similar clustering within replicates of the same EOAD samples but worse clustering for the NDC samples; this could suggest variable differentiation of clones within patient samples as well as across samples during the iPSCs differentiation process into neurons (Fig. 1F, right). Furthermore, examining heatmap and dendrogram clustering of the overall expression profile of the filtered genes (*n* = 21,546) demonstrates that the expression patterns from both EOAD and control neurons did not show apparent differences, with no distinct patterning observed between the two groups (Fig. 1G).

To assess the neuronal differentiation state of the EOAD and NDC neurons, we first focused on genes that regulate neuron lineage (curated from GO: Biological Process and Reactome genesets, *n* = 1078). MDS revealed a slightly greater variance within the EOAD and NDC conditions but no visible separation between the two (Fig. 1H, left). When performing UMAP analysis, while we observe a single cluster of the majority of  EOAD patient clones, there is greater separation of NDC clones  within and between NDC subjects overall, suggesting a variable neuron lineage state across all samples (Fig. 1H, right). This suggests that iPSC-derived EOAD neurons do not show a similar separation from healthy neurons as observed in familial Alzheimer’s disease (FAD) neurons with *PSEN1*, *PSEN2*, and *APP* mutations [4, 5].

Next, we sought to assess the expression profile of these neurons based on only neuron lineage genes. We saw a similar trend to the findings reported above: no noticeable gene expression differences observed in key marker genes for both EOAD and NDC neurons (Fig. 1I, left). As such, gene expression differences between NDC and EOAD were not well captured under the conditions of our study. We then observed a subset of these neuron lineage genes that mainly regulate post-mitotic neuron maturation (*NEFL, SOX2, UCHL1, TUBB3, MAP2, DCX, NEUROD1* and *APOE*) [9–13] (Fig. 1I, right). These genes were selected since they are the most common markers for post-mitotic neurons found in literature [9–13] and we observe similar expression profiling for both NDC and EOAD neurons.

Since neither EOAD nor NDC samples clustered together, we next assessed whether EOAD and NDC neurons have established a mature neuronal identity [14] or whether there may be alternative cell lineages in our cultures by first looking at different neural lineage stages ranging from neural progenitors, glia, and neurons (immature and mature) derived from human post-mortem brain and patient-derived iPSC single-cell mean expression data [9, 12, 15–18]. When we interrogated marker genes specific to these major cell types via gene-level expression across EOAD and NDC neurons, we observed the highest average marker gene expression associated with neurons, followed by glial and progenitor cells, but without statistically significant expression differences between EOAD and NDC (Additional file 1: Fig. S1A). When using all marker genes, we observe a slight decrease in average gene-level expression when comparing expression data from all EOAD relative to all NDC neurons (Additional file 1: Fig. S1A). It is worth noting that the potential existence of populations of these earlier lineage cell types could contribute to the relative variability seen in our iPSC-derived EOAD and NDC neuron cultures. We then proceeded to look at the average gene-level expression of different EOAD and NDC subject lines using cell type markers from categories such as progenitor cells, glial cells, immature neurons, and mature neurons (Additional file 1: Fig. S1B–E) and cell subcategories such as neural progenitor cells (NPCs), oligodendrocytes, excitatory neurons, and inhibitory neurons (Additional file 1: Fig. S2D–G). For all cell types and subcategories, we find no significant differences between EOAD and NDC neurons (Additional file 1: Figs. S1B–E, S2D-G). Next, we performed clustering in MDS space within the different cell type classes to see if the separation of the two conditions changed with marker gene expression of different stages of neuronal lineage; we observed tighter clustering within experimental condition (EOAD or NDC) despite slight overlap for glial cells (Additional file 1: Fig. S1G) followed by mature neurons (Additional file 1: Fig. S1I), immature neurons (Additional file 1: Fig. S1H), and progenitor cells (Additional file 1: Fig. S1F). Furthermore, we also observe an increase in the separation of EOAD and NDC neurons along dimension 1 in MDS space for mature neuron marker genes (Additional file 1: Fig. S1I), providing evidence of the heterogeneity involved. Looking closely at different groups of cellular subtype markers (Additional file 1: Fig. S2A–C) across different EOAD patients relative to all NDC subjects, we observe no significant expression differences in any cellular subtype (Additional file 1: Fig. S2D–G). When we observe these cellular subtypes in MDS space, there is tighter clustering involved within the experimental condition, despite the slight overlap in marker gene expression for neural progenitor cells (Additional file 1: Fig. S2H) and oligodendrocytes (Additional file 1: Fig. S2I) when compared to excitatory and inhibitory neurons (Additional file 1: Fig. S2J, K) which are clustered more sparsely. This suggests that heterogeneity increases as EOAD and NDC iPSCs progress to a more mature state characterized by excitatory and inhibitory neurons.

Using a standard protocol for making human iPSC-derived neurons, we have not effectively discriminated between NDC and EOAD neurons. Our findings suggest that this approach for examining the biology of EOAD may fail either to adequately differentiate EOAD and NDC neurons or capture changes in gene expression characteristic of AD. The lack of patient clustering and variance observed in the EOAD study may be because diverse etiological factors contributing to aging-associated epigenetic changes (e.g., RNA modifications and non-coding RNA regulation) [19] are not preserved during iPSC reprogramming [20]. We also investigated the expression levels of marker genes associated with major alternative cell types and their cell subtypes to determine whether they contribute to the EOAD and NDC neuron cell culture; we quantified average expression levels and MDS clustering with no adjustments made to account for the presence of variable neuronal populations, which revealed an increasing separation between EOAD and NDC cultures for mature neuron marker genes. Furthermore, we can suggest that the differentiation protocol via SMAD inhibition and bFGF removal used to generate the EOAD and NDC neurons did not ultimately result in a purely differentiated mature neuron population. It is likely that there is a mix of different neuronal lineages, including those from an earlier lineage state (more specifically progenitor cells) and glial cells (not necessarily representative of oligodendrocytes, but likely representative of astrocytes, microglia and OPCs), but it is unclear their relative contribution to the neuron culture. As such, we were not able to capture the phenotype differences between EOAD and NDC neurons. This is due to the following: (1) the variation of differentiation across clones of patients, (2) the variability within EOAD patients relative to NDC subjects and (3) the relative immature state of the neuron cultures.

Methods that preserve the epigenetic signatures may provide better models for studying sporadic AD. It is likely that induced neurons (iNs) that undergo direct neuronal conversion from fibroblasts via small molecule reprogramming [21] can capture a more mature neuron state of the EOAD brain and merit further studies. Previous studies have shown that age-dependent cellular programs of patients with late-onset sporadic AD derived from direct iNs are characterized by downregulation of mature neuronal properties (i.e., loss of mature neuronal fate, neuronal dedifferentiation) and upregulation of cell cycle re-entry. Additionally, age-related changes in the epigenetic landscape appear to underlie a hypo-mature neuron state in iNs, thus directing toward a more de-differentiated state of sporadic AD [14, 20]. Ultimately, utilizing an iN model may be required to preserve both EOAD gene expression and epigenetic signatures, thus informing the cellular environment required to understand the neuronal biology of EOAD.

## Methods

### iPSC neuron generation

Fibroblasts were obtained by skin biopsy (n = 4 early-onset Alzheimer’s disease (EOAD) patients composed of 2 females and 2 males; n = 5 non-demented control (NDC) subjects composed of 4 females and 1 male) at the Shiley-Marcos Alzheimer’s Disease Research Center at the University of California, San Diego (UCSD) in accordance with UC San Diego IRB approval. The age at onset (AAO) of EOAD patients ranged from 51–56 years, whereas NDC subjects ranged from 76–82 years. Human dermal fibroblasts were grown on a Trevigen Reduced Growth Factor Basement Membrane Extract (Trevigen Cat. No. 3433-005-01) with Stem Cell mTeSR-1 media (STEMCELL Technologies Cat. No. 05851) for 5 weeks. Then they were reprogrammed into iPSCs using Sendai Virus [22] from the 2.0 Sendai Virus Kit (Life Tech Cat. No. A16517) at the Salk Institute Stem Cell Core. Direct differentiation of iPSCs to a population of neurons was performed as previously described [6]. Briefly, iPSCs were differentiated into neurons using two stages: First, iPSCs were differentiated into neural pluripotent cells (NPCs) using dual Suppressor of Mothers Against Decapentaplegic (SMAD) inhibitors SB431542 (StemRD No. 50176030; Final concentration = 10 uM), LDN193189 (BioVision No. 1995–5; Final concentration = 0.5 uM), and the recombinant protein Noggin (R&D Systems No. 1967-NG/CF; Final concentration = 0.5 ug/ml)) in 500 ml of Neural Maintenance Media (NMM) based on previous methods [6, 23]. Next, NPCs were differentiated into mixed neuronal cell populations by basic-Fibroblast Growth Factor (bFGF) (Millipore Cat. No. GF003AF; Final concentration = 20 ng/ml) withdrawal [24] for 3–4 weeks. Neurons were suspended in neuronal media and 150,000–2 million cells were counted, pelleted down and then flash-frozen into individual tubes to retain cellular integrity long-term. One male NDC subject developed mild cognitive impairment (MCI) later in life and thus was excluded from further downstream RNA-seq analysis.

### RNA extraction

Total RNA was extracted from previously-harvested EOAD and NDC frozen neuron pellets (replicates, n = 3) with cell counts ranging from 500,000–2 million cells using the Rneasy Plus Micro Kit protocol (Qiagen, catalog no. 74034) as previously described [4, 25]. Concentrations of total RNA were determined using the Nanodrop 2000c according to the manufacturer’s guidelines. QC measurements were performed at the UC San Diego IGM sequencing core to evaluate the RNA Integrity Numbers (RIN) using TapeStation (Agilent Technologies), which ranged from 7.8–9.8.

### RNA sequencing

Libraries were generated for RNA-seq using the Illumina Ribo-Zero Plus rRNA Depletion kit with IDT for Illumina RNA UD Indexes (Illumina, San Diego, CA). Samples were processed following manufacturer’s instructions. Resulting libraries were multiplexed and sequenced on an Illumina NovaSeq 6000 generating paired-end, 100-bp (PE100) to a depth of approximately 25 million reads per sample at the UC San Diego IGM sequencing core. In addition, samples were demultiplexed using the *bcl2fastq* v.2.20 Conversion Software (Illumina, San Diego, CA). 

### RNA-seq data processing and clustering

Preprocessing of the paired-end RNA-seq data was conducted using the Trimgalore! package v0.6.4 by removing adapters and low quality reads using CutAdapt v1.18 [26] with the following options: -quality 25 -fastqc -illumina -length 98 -paired. Trimmed RNA-seq reads were then mapped to the GRCh38.104 human transcriptome using Kallisto v0.46.1 [27] with the following options: -bias -rf-stranded -b 100. Transcript abundances from Kallisto were imported and summarized to the gene level using *tximport* v1.22.0 [28]. A DGEList object was created from gene-level read counts using the *DGEList* function from *edgeR* v3.36.0 [29]. Lowly expressed genes were filtered out using *filterByExpr* function in *edgeR.* Then gene-level counts were normalized using the weighted mean trimmed of M values (TMM) in the *calcNormFactors* R package. Normalized, filtered counts were used for differential gene expression (DGE) analysis using the *limma* v3.50.1 [30] R package. For multi-dimensional scaling (MDS) analysis, the filtered expression counts were corrected for experimental condition, sex, and sequencing run batch using the *removeBatchEffect* function within *limma*, samples were grouped accordingly, and plotted using the *glimmaMDS* function in the *Glimma* v2.4.0 [31] R package. Additional unsupervised clustering analyses were performed, such as uniform manifold approximation and projection (UMAP) using *umap* v0.2.10.0 [32] based on the following parameters: (1) metric which computes different distance metrics in high dimensional space based on: (a) cosine and (b) pearson2 that only relies on centering and (2) n_neighbors = 3 that allows local data to only be preserved. Differential gene expression (DGE) analysis for relative comparison of all EOAD and NDC subjects were performed using the the *kmFit* function within *kimma* based on a linear mixed model [33] represented by sex, sequencing batch, and experimental condition covariates with an added random effect by patient number identification. Differentially expressed genes (DEGs) from a filtered gene list were defined using a false discovery rate (FDR)-adjusted-p-value cutoff of < 0.05 from the mixed effects model as a contrast between EOAD patients relative to NDC subjects using *lme.contrast* function from the *kimma* R package. Quasi-proportional Venn diagrams of DEG overlap between the FAD mutations were generated using the *nVennR* v0.2.3 package in R [34].

### RNA-seq data expression profiling

To compare filtered gene-level count expression based on (1) normalized, filtered genes and (2) genes that regulate neuron lineage (gene list sourced from literature) between paired-end EOAD relative to NDC, z-score normalization was applied. After filtered counts were acquired, they were either (1) converted to z-scores using the *scale* function from *base* v4.1.3 [35] or (2) subset to only neuron lineage genes using the *merge* function by common gene symbols and then underwent z-score conversion using *scale* where the parameters center = TRUE and scale = TRUE were set. Finally, hierarchical agglomerative clustering using the Ward method [36] from the *hclustfun* parameter within the *heatmap* function from the *stats* [37] R package was performed to visually represent the set of z-scores. The mean z-scores per EOAD or control subject were calculated as initial input prior to performing the clustering.

### RNA-seq data classification surrogate neuron marker analysis

To determine the relative abundance of the sequenced reads (i.e. counts) at the gene-level (in transcripts per million, TPM) using both the *tximport* and *filterbyExpr* R packages for the EOAD study neurons (4 diseased patient and 4 healthy subject neurons) across different cell type proportions, we subset the reads according to markers from different cellular classes (i.e. progenitor cells, glial cells, immature and mature neurons) and subtypes (early, radial glial, intermediate and neural progenitor cells [IPCs and NPCs], astrocytes, microglia, oligodendrocyte precursor cells [OPCs], oligodendrocytes, excitatory, inhibitory and glycolytic neurons) from human 10x  single-cell data originating from multiple sources such as the antibody database, ABCAM [9], additional literature for excitatory neurons from a single-nucleus RNA sequencing dataset that selectively characterized for excitatory neurons from postmortem brains spanning from early to late AD progression [38], immature neurons from a review about neurogenesis in the human hippocampal dentate gyrus [12] and glycolytic neurons from different human, post mortem AD brain regions and patient-derived AD iPSCs [15–17] and then finally the Allen Brain Atlas, particularly from the primary motor cortex, M1 brain region with a trimmed mean expression value threshold > 5 [18]. After, we gathered a list of known differentially expressed up-regulated and down-regulated genes (DEGs) to be expressed in the various cell types by merging the genes with the filtered read counts (n = 21,546 genes) using the *merge* function by common gene symbols. We were then able to obtain the following number of genes for each cell type (n = 358 genes for astrocytes; n = 140 genes for microglia; n = 2041 genes for oligodendrocytes; n = 469 genes for OPCs; n = 48 genes for progenitor cells subset to n = 4 genes for early, n = 18 genes for radial glia, n = 11 genes for IPC and n = 19 genes for NPC; n = 2242 genes for glial cells; n = 9 genes for mature neurons; n = 14 genes for immature neurons; n = 5228 genes for excitatory neurons; n = 4751 genes for inhibitory neurons; and then n = 98 genes for glycolytic neurons). Then we calculated the mean gene-level read counts per experimental condition for the following: (1) all filtered neurons, (2) progenitor cells, (3) glial cells, (4) immature neurons, and (5) mature neurons only genes. Code for all analysis is available at https://github.com/SubramaniamLab/EOAD-RNA-seq-Manuscript and 10.5281/zenodo.8320537.

### Supplementary Information

**Additional file 1: Figure S1. A.** Bar plot showing average gene counts normalized to read library size (TPM) for all filtered neurons, progenitor cells, glial cells, immature neurons, and mature neurons across NDC and EOAD neurons. **B-E.** Bar plot showing average gene counts normalized to read library size (TPM) across different groups of **B** progenitor cells, **C** glial cells, **D** immature neurons and **E** mature neurons marker genes across NDC and EOAD neurons. **F-I.** Multi-dimensional scaling (MDS) analysis after batch correction by experimental condition, sex and sequencing batch of filtered normalized RNA-seq data subset to **F** progenitor cells, **G** glial cells, **H** immature neurons and **I** mature neurons genes. **Figure S2. A-C.** Pie chart distribution of cellular subtypes classified in** A** progenitor cells, **B** glial cells and **C** neurons. **D-G.** Bar plot showing average gene counts normalized to read library size (TPM) across different groups of different **D** neural progenitor cells, **E** oligodendrocytes, **F** excitatory neurons and **G** inhibitory neurons marker genes across NDC and EOAD neurons. **H–K.** Multi-dimensional scaling (MDS) analysis after batch correction by experimental condition, sex and sequencing batch of filtered normalized RNA-seq data subset to **H** neural progenitor cells, **I** oligodendrocytes, **J** excitatory neurons and **K** inhibitory neurons genes. **Figure S3. A.** RNA-seq volcano plots of differentially expressed genes (DEGs) across different EOAD patients relative to all NDCs as determined by *kimma* with an FDR p-value < 0.05.

## Data Availability

RNA-seq data is available at the NCBI GEO under the accession GSE231341.

## Code availability

Code for all analysis is available for download from https://github.com/SubramaniamLab/EOAD-RNA-seq-Manuscript and https://doi.org/10.5281/zenodo.8320537.

## References

[1] Mendez MF. Early-onset Alzheimer Disease and Its Variants. Continuum (Minneap Minn). 2019;25:34–51. 10.1212/CON.0000000000000687 PMC6538053 30707186

[2] Sirkis DW, Bonham LW, Johnson TP, La Joie R, Yokoyama JS (2022). Dissecting the clinical heterogeneity of early-onset Alzheimer’s disease. Mol Psychiatry.

[3] Caldwell AB, Anantharaman BG, Ramachandran S, Nguyen P, Liu Q, Trinh I (2022). Transcriptomic profiling of sporadic Alzheimer’s disease patients. Mol Brain.

[4] Valdes P, Caldwell A, Liu Q, Fitzgerald M, Ramachandran S, Karch C, et al. Integrative multiomics reveals common endotypes across PSEN1, PSEN2, and APP mutations in familial Alzheimer’s disease. Research Square. 2022;1-30. 10.21203/rs.3.rs-2356131/v1 10.1186/s13195-024-01659-6 PMC11699654 39754192

[5] Caldwell AB, Liu Q, Schroth GP, Galasko DR, Yuan SH, Wagner SL (2020). Dedifferentiation and neuronal repression define familial Alzheimer’s disease. Sci Adv.

[6] Shi Y, Kirwan P, Livesey FJ (2012). Directed differentiation of human pluripotent stem cells to cerebral cortex neurons and neural networks. Nat Protoc.

[7] Henneges C, Reed C, Chen Y-F, Dell’Agnello G, Lebrec J. Describing the sequence of cognitive decline in alzheimer’s disease patients: results from an observational study. J Alzheimers Dis. 2016;52:1065–80. 10.3233/JAD-150852 PMC4927893 27079700

[8] Davis DH, Creavin ST, Yip JL, Noel-Storr AH, Brayne C, Cullum S (2015). Montreal cognitive assessment for the diagnosis of Alzheimer’s disease and other dementias. Cochrane Database Syst Rev.

[9] Neuronal, neural stem cell and glial cell markers | Abcam. https://www.abcam.com/neuroscience/neural-markers-guide. Accessed 29 Aug 2023.

[10] CNS Cell Markers | GeneTex. Available from: https://www.genetex.com/Research/Overview/neuroscience/CNS_cell_markers. Accessed 11 Apr 2023.

[11] Gatt A, Lee H, Williams G, Thuret S, Ballard C (2019). Expression of neurogenic markers in Alzheimer’s disease: a systematic review and metatranscriptional analysis. Neurobiol Aging.

[12] Hagihara H, Murano T, Ohira K, Miwa M, Nakamura K, Miyakawa T (2019). Expression of progenitor cell/immature neuron markers does not present definitive evidence for adult neurogenesis. Mol Brain.

[13] Neuronal and Glial Cell Markers. Cell signaling technology. https://www.cellsignal.com/pathways/neuronal-and-glial-cell-markers. Accessed 11 Apr 2023.

[14] Mertens J, Herdy JR, Traxler L, Schafer ST, Schlachetzki JCM, Böhnke L (2021). Age-dependent instability of mature neuronal fate in induced neurons from Alzheimer’s patients. Cell Stem Cell.

[15] Qiu Z, Bai X, Ji X, Wang X, Han X, Wang D (2022). The significance of glycolysis index and its correlations with immune infiltrates in Alzheimer’s disease. Front Immunol.

[16] Saito ER, Miller JB, Harari O, Cruchaga C, Mihindukulasuriya KA, Kauwe JSK (2021). Alzheimer’s disease alters oligodendrocytic glycolytic and ketolytic gene expression. Alzheimer Dementia.

[17] Ryu W-I, Bormann MK, Shen M, Kim D, Forester B, Park Y (2021). Brain cells derived from Alzheimer’s disease patients have multiple specific innate abnormalities in energy metabolism. Mol Psychiatry.

[18] Gilbert TL (2018). The Allen brain atlas as a resource for teaching undergraduate neuroscience. J Undergrad Neurosci Educ.

[19] Wang K, Liu H, Hu Q, Wang L, Liu J, Zheng Z (2022). Epigenetic regulation of aging: implications for interventions of aging and diseases. Sig Transduct Target Ther.

[20] Zhou C, Ni W, Zhu T, Dong S, Sun P, Hua F (2022). Cellular reprogramming and its potential application in Alzheimer’s disease. Front Neurosci.

[21] Mertens J, Paquola ACM, Ku M, Hatch E, Böhnke L, Ladjevardi S (2015). Directly reprogrammed human neurons retain aging-associated transcriptomic signatures and reveal age-related nucleocytoplasmic defects. Cell Stem Cell.

[22] Schlaeger TM, Daheron L, Brickler TR, Entwisle S, Chan K, Cianci A (2015). A comparison of non-integrating reprogramming methods. Nat Biotechnol.

[23] Chambers SM, Fasano CA, Papapetrou EP, Tomishima M, Sadelain M, Studer L (2009). Highly efficient neural conversion of human ES and iPS cells by dual inhibition of SMAD signaling. Nat Biotechnol.

[24] Qian L, Tcw J (2021). Human iPSC-based modeling of central nerve system disorders for drug discovery. Int J Mol Sci.

[25] Loontiens S, Depestel L, Vanhauwaert S, Dewyn G, Gistelinck C, Verboom K (2019). Purification of high-quality RNA from a small number of fluorescence activated cell sorted zebrafish cells for RNA sequencing purposes. BMC Genomics.

[26] Taking appropriate QC measures for RRBS-type or other -Seq applications with TrimGalore! Babraham Bioinformatics. 2019; https://github.com/FelixKrueger/TrimGalore/blob/master/Docs/Trim_Galore_User_Guide.md.

[27] Bray NL, Pimentel H, Melsted P, Pachter L (2016). Near-optimal probabilistic RNA-seq quantification. Nat Biotechnol.

[28] Soneson C, Love MI, Robinson MD (2016). Differential analyses for RNA-seq: transcript-level estimates improve gene-level inferences. F1000Res.

[29] Robinson MD, McCarthy DJ, Smyth GK (2009). edgeR: a Bioconductor package for differential expression analysis of digital gene expression data. Bioinformatics.

[30] Ritchie ME, Phipson B, Wu D, Hu Y, Law CW, Shi W (2015). limma powers differential expression analyses for RNA-sequencing and microarray studies. Nucleic Acids Res.

[31] Su S, Law CW, Ah-Cann C, Asselin-Labat M-L, Blewitt ME, Ritchie ME (2017). Glimma: interactive graphics for gene expression analysis. Bioinformatics.

[32] McInnes L, Healy J, Melville J. UMAP: uniform manifold approximation and projection for dimension reduction. arXiv; 2020. Available from: http://arxiv.org/abs/1802.03426. Accessed 18 Mar 2023.

[33] Dill-McFarland KA, Mitchell K, Batchu S, Segnitz RM, Benson B, Janczyk T (2023). Kimma: flexible linear mixed effects modeling with kinship covariance for RNA-seq data. Bioinformatics.

[34] Pérez-Silva JG, Araujo-Voces M, Quesada V (2018). nVenn: generalized, quasi-proportional Venn and Euler diagrams. Bioinformatics.

[35] base-package: The R Base Package. https://rdrr.io/r/base/base-package.html. Accessed 18 Mar 2023.

[36] Murtagh F, Legendre P (2014). Ward’s hierarchical agglomerative clustering method: which algorithms implement ward’s criterion?. J Classif.

[37] stats-package: The R Stats Package. https://rdrr.io/r/stats/stats-package.html. Accessed 18 Mar 2023.

[38] Leng K, Li E, Eser R, Piergies A, Sit R, Tan M (2021). Molecular characterization of selectively vulnerable neurons in Alzheimer’s disease. Nat Neurosci.

