# Supplementary figures and images for: Limitations of the human iPSC-derived neuron model for early-onset Alzheimer’s disease
Source: Mol Brain. 2023 Nov 3;16:75. doi: 10.1186/s13041-023-01063-5 (PMC10623777; doi:10.1186/s13041-023-01063-5)

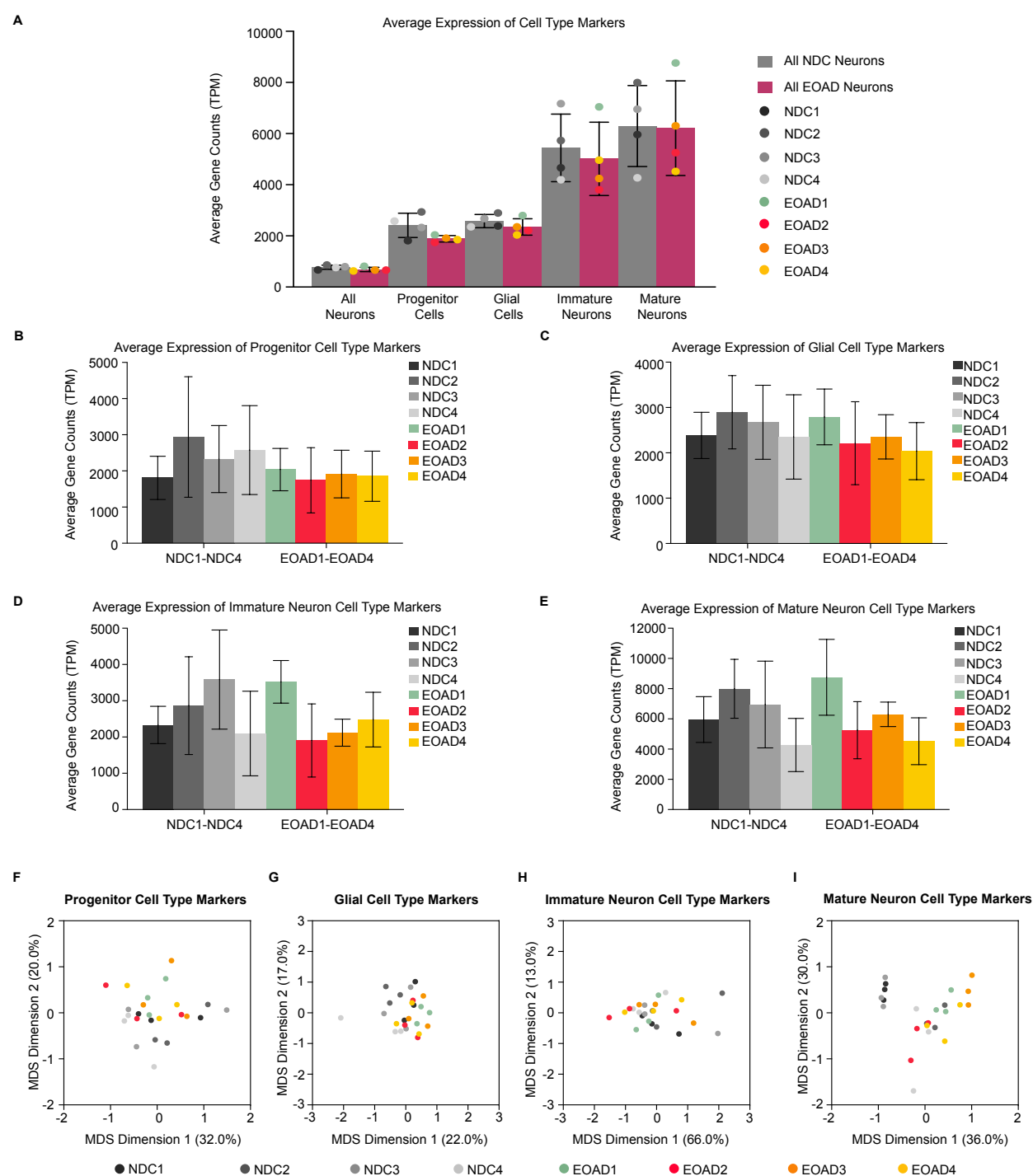

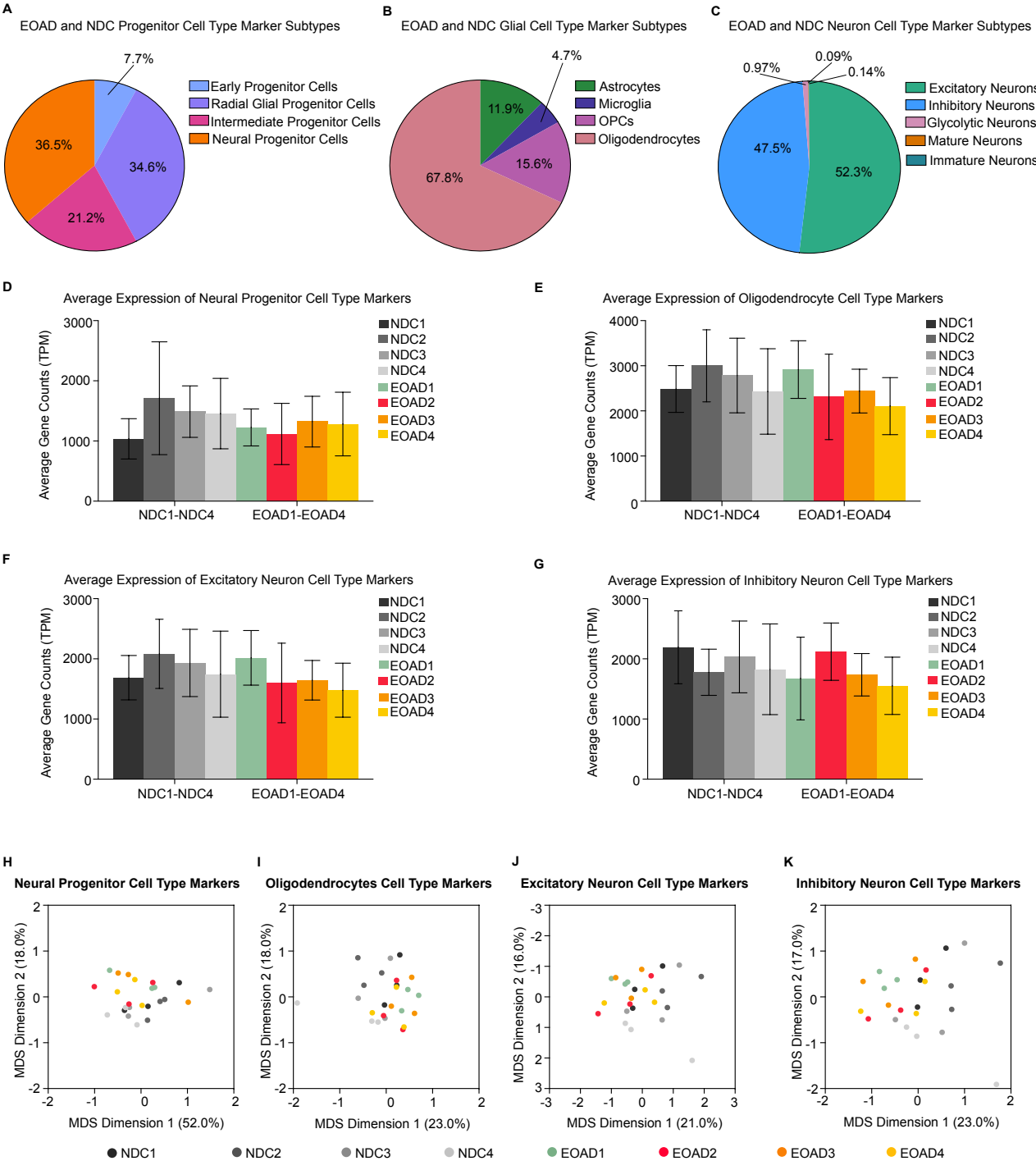

A

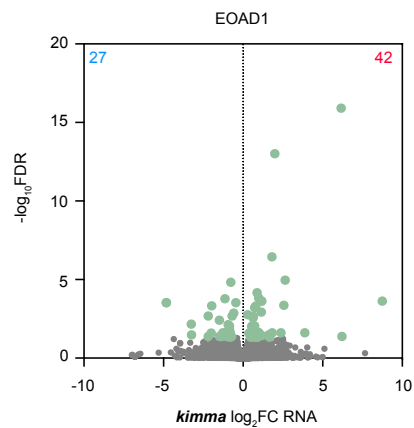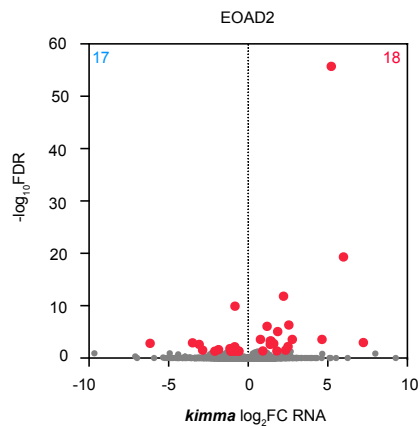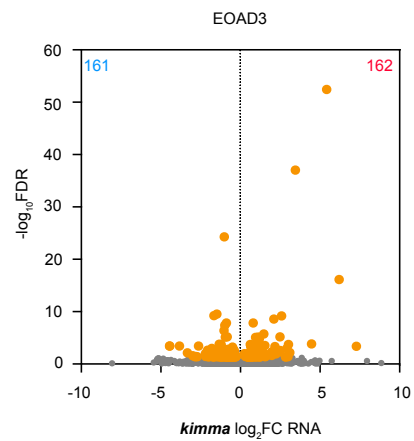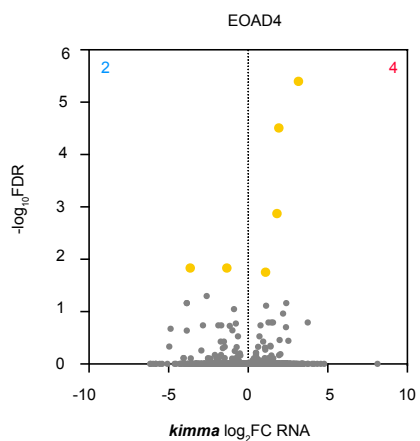

Supplement: Supplementary file 1 — Additional file 1: Figure S1. A. Bar plot showing average gene counts normalized to read library size (TPM) for all filtered neurons, progenitor cells, glial cells, immature neurons, and mature neurons across NDC and EOAD neurons. B-E. Bar plot showing average gene counts normalized to read library size (TPM) across different groups of B progenitor cells, C glial cells, D immature neurons and E mature neurons marker genes across NDC and EOAD neurons. F-I. Multi-dimensional scaling (MDS) analysis after batch correction by experimental condition, sex and sequencing batch of filtered normalized RNA-seq data subset to F progenitor cells, G glial cells, H immature neurons and I mature neurons genes. Figure S2. A-C. Pie chart distribution of cellular subtypes classified in A progenitor cells, B glial cells and C neurons. D-G. Bar plot showing average gene counts normalized to read library size (TPM) across different groups of different D neural progenitor cells, E oligodendrocytes, F excitatory neurons and G inhibitory neurons marker genes across NDC and EOAD neurons. H–K. Multi-dimensional scaling (MDS) analysis after batch correction by experimental condition, sex and sequencing batch of filtered normalized RNA-seq data subset to H neural progenitor cells, I oligodendrocytes, J excitatory neurons and K inhibitory neurons genes. Figure S3. A. RNA-seq volcano plots of differentially expressed genes (DEGs) across different EOAD patients relative to all NDCs as determined by kimma with an FDR p-value < 0.05. [file 13041_2023_1063_MOESM1_ESM.pdf]
